# Achieving Rapid Healing and Low Complication Rates in Patellar Fracture Fixation: The Benefits of Cerclage and Figure-of-Eight Configuration

**DOI:** 10.7759/cureus.39059

**Published:** 2023-05-15

**Authors:** Avinash Kachare, Jairam Jagiasi, Pravin Jadhav, Kishor Munde

**Affiliations:** 1 Department of Orthopaedics, Lokmanya Tilak Municipal Medical College and Lokmanya Tilak Municipal General Hospital, Mumbai, IND

**Keywords:** patella reconstruction, extensor mechanism, k wire tbw, patella fracture, tension band wiring

## Abstract

Background and objective

Patellar fractures account for around 1% of all fractures. Conservative treatment is advised in patients without any incompatibility of articular surfaces or those with intact extensor mechanisms. More than a 2-mm articular gap due to fracture warrants surgical intervention. Tension band wiring (TBW) is a commonly used practice for fixation, However, there is still controversy about its effectiveness and complications arising due to the hardware. Modification of this technique by using K-wires has been considered a method of choice, but this technique is associated with complications due to K-wires. The Pyrford technique is a method for patellar fracture fixation by circumferential cerclage and anterior TBW. We employed the figure-of-eight configuration over the circumferential wire. This study aimed to analyze the outcomes of TBW of the patella without K-wires by assessing the rate of complication and functional outcomes.

Materials and methods

A total of 38 patients with OTA 34C type, simple and comminuted type of patella fractures aged between 22 and 70 years were treated with circumferential cerclage and figure-of-eight TBW. All patients underwent patellar fixation with cerclage and through direct purchase of SS wire via quadriceps and patellar tendon. Patients were followed up for one to three years. We analyzed differences in the range of motion, fracture reduction, fracture healing time, Bostman score for knee function, and complications.

Results

The mean age of the patients was 45 years. After TBW without K-wires, fracture healing and functional outcomes were satisfactory according to patient feedback and clinocoradiological examinations. Of note, 35 out of 38 patients (92%) had gained up to 90 degrees of active flexion at the end of one week. One patient (2.42%) developed a superficial infection. All fractures had achieved union at the end of 16 weeks. Malunion or nonunion was not noted in any of the cases. There was no case of implant removal. The average Bostman score at the 12-month follow-up was 28.5 ±1.5. The incidence of complications due to K-wire was nullified.

Conclusion

Based on our findings, the described method leads to better functional outcomes, decreasing hardware-related complications, and can be used in simple as well as comminuted fractures. The fracture healing and functional outcomes and rate of complications were satisfactory.

## Introduction

As the largest sesamoid bone in the body and a key component of the extensor mechanism formed by the quadricep tendon and patellar tendon, the patella plays a crucial role in locomotion. Its anatomical position provides a biomechanical advantage by increasing the moment arm of the quadriceps, resulting in a 30% increase in extension strength with a gliding action across the trochlea [1]. However, at full flexion of the knee, the force on the patella reaches 7.65 times the body weight, and at 60 degrees, it is four times the body weight [2]. As the patella is located in a high mechanical force transmission zone, methods for the fixation of patellar fractures must be sufficient enough to counteract the distracting forces at the site of extensor mechanism disruption. Various methods have been used to repair patellar extensor mechanisms, including modified tension band wiring (TBW), AO TBW [3], the Pyrford technique [4], which combines cerclage wiring and the anterior tension band principle, and buttress plate fixation for comminuted fractures [5].

Patellar fractures are relatively rare, accounting for approximately 1% of all fractures [6]. Surgical intervention is typically indicated when there is an articular step-off of 2 mm or more, or in cases of loss of the extensor mechanism in the knee [7]. TBW is a commonly employed fixation technique; however, the use of hardware is associated with potential complications. While modification of the TBW technique using K-wires has been proposed as a method of choice [3], biological and hardware-related complications, such as K-wire migration, wound infection, pain, arthrofibrosis, and patellofemoral joint irritation, have been observed [8,9]. Additionally, fixation with K-wires has been associated with a higher likelihood of implant removal compared to screw fixation [10].

In our study, we used a figure-of-eight configuration rather than a circumferential wire to evaluate the outcomes of TBW for patellar fractures without K-wire anchorage. Our aim was to assess the rate of complications and functional outcomes associated with this technique. This technique has the potential to reduce the risk of complications associated with K-wire anchorage, but there is currently limited research available on its outcomes and efficacy.

## Materials and methods

**Figure 1:**
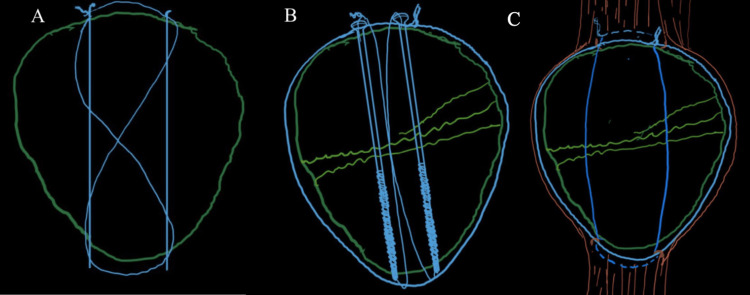
Commonly used methods of patella fracture fixation (A) AO method of tension band wiring. (B) Tension band wiring of the patella with cannulated screws. (C) Pyrford technique

Figure 1 shows the commonly used methods of patella fracture fixation.

**Figure 2:**
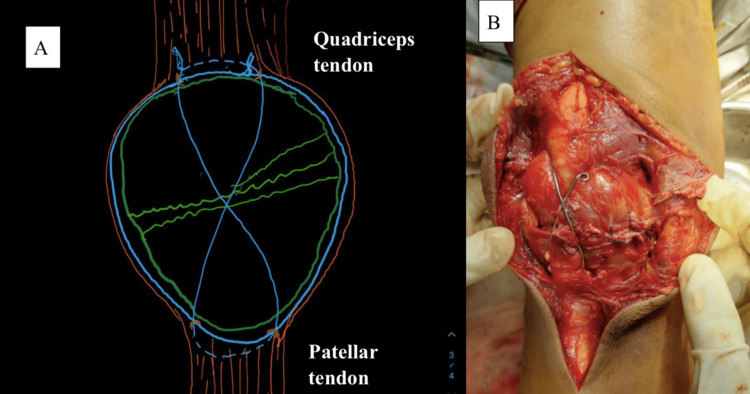
(A) Image depicting our method of patellar fixation with cerclage and through direct purchase of SS wire via quadriceps and patellar tendon. (B) Intraoperative clinical image showing fixation of patella fracture with our method

This retrospective study included 38 patients with OTA type 34C patellar fracture, of which 21 were males and 17 were females [11]. All fractures were caused by a direct blow to the patella following a road traffic accident. Under general or spinal anesthesia, the patients underwent surgical intervention with an incision made over the patella to visualize the extensor aponeurosis and identify the tear caused by the fracture. The patellar fragments were cleaned, and the hematoma was removed. To fixate the fracture, an 18-gauge stainless steel wire was used to encircle the patella by passing through the quadriceps tendon, retinaculum, and patellar tendon. The wire ends were tightened and tied under tension at the superolateral corner of the patella. Another 18-gauge stainless steel wire was passed in a figure-of-eight configuration through direct purchase in the quadriceps tendon and patellar tendon (Figure 2). A medial arthrotomy was performed to confirm the reduction by palpating the joint surface underneath and radiologically. The range of motion was checked under an image intensifier to ensure adequate reduction during flexion. The postoperative protocol for patella fracture fixation with TBW includes weight-bearing as tolerated from the first postoperative day, range-of-motion exercises and quadriceps strengthening from the first postoperative week, and physiotherapy. Follow-up appointments were scheduled at four, eight, and 16 weeks after surgery to monitor patient progress.

## Results

We carried out follow-up research on 38 patients with OTA type 34C patella fractures. The mean age of the patients was 45 years; patients were followed up for a period of 12-36 months. At the end of the first week, 92% (35/38) of patients achieved a range of motion of 90 degrees, with the remaining seven patients showing extension lag that was later improved with vigorous physiotherapy. Knee stiffness was observed in two patients, which was managed by continuous passive movement and quad strengthening with static and dynamic methods, resulting in the desired range of motion after six weeks.

**Figure 3:**
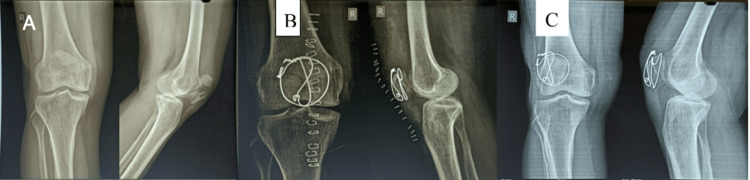
(A) Preoperative X-rays. (B) Immediate postoperative X-rays. (C) Follow-up X-rays at 16 weeks

**Figure 4:**
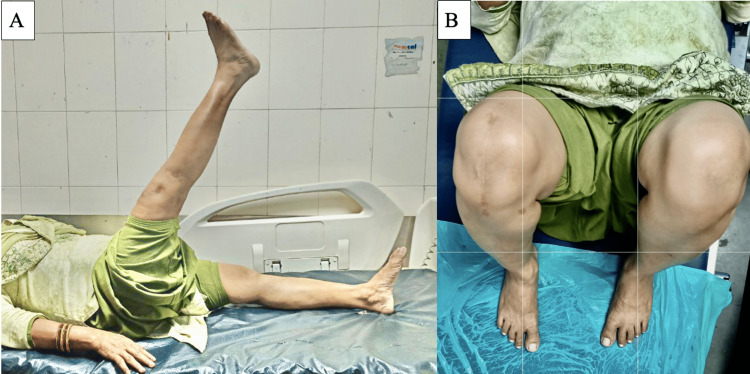
A and B: Images showing clinical follow-up of a patient with active straight leg raising test and full knee range of motion at 16 weeks

**Figure 5:**
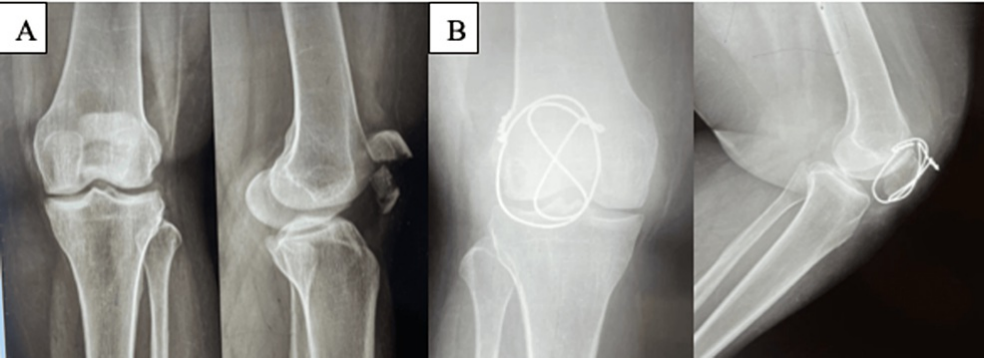
(A) Preoperative X-rays. (B) Follow-up X-rays showing the complete union of patella fracture at 16 weeks

**Figure 6:**
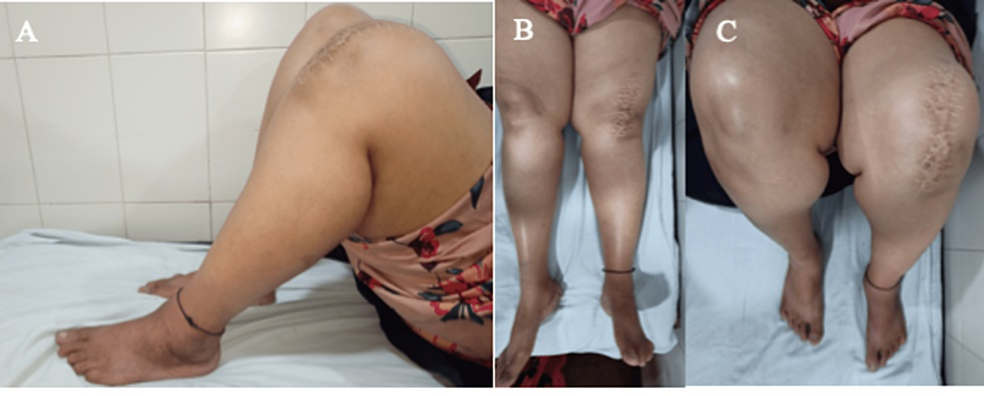
A, B, and C: Images showing clinical follow-up of the patient shown in Figure 5

**Figure 7:**
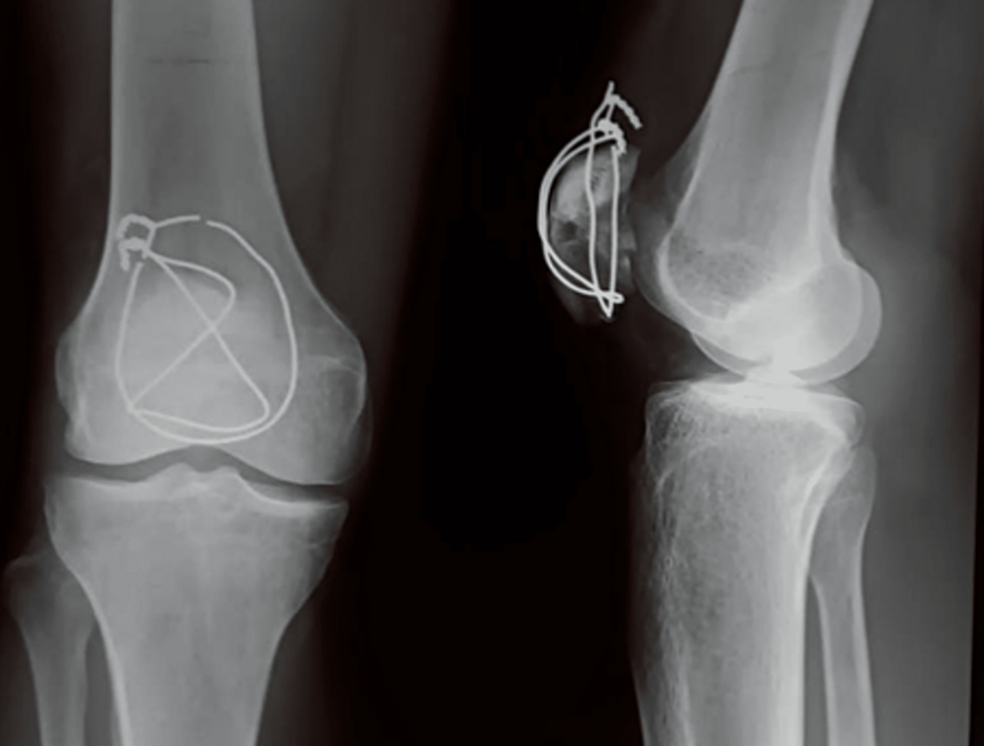
X-ray showing broken cerclage wire at 18 months of follow-up in one case

All fractures achieved complete union by the end of 16 weeks. The long-term functional and rehab outcomes were assessed by using Bostman scores at 12 months of follow-up (Figures 3-6). The average Bostman score was 28.5 ±1.5, and the average flexion-extension was 124 and 1.3 degrees. Only one patient (2.42%) developed a superficial infection, which was treated with broad-spectrum antibiotics and did not affect the fracture healing time. In the second year of follow-up, one case (2.42%) was observed with a broken SS wire at the superolateral corner (Figure 7). However, there was no case of malunion or nonunion, and no implant removal was required over the course of one to three years. Notably, the incidence of complications due to K-wires was nullified in our study.

## Discussion

A patellar fracture is a challenging condition to manage surgically due to the presence of multiple small fragments and the patella's role in the extensor mechanism, which presents difficulties in rehabilitation [12]. Cerclage wiring fixation is commonly used in combination with other methods of fixation [13]. However, Matsuo et al. have reported achieving and maintaining reduction by utilizing the soft tissue around the patella, resulting in zero implant-related complications, but they noted a high rate of nonunion [14]. In this study, we utilized cerclage with a figure-of-eight configuration to minimize hardware while achieving desirable functional outcomes.

Patellar fracture healing occurs relatively fast as it involves a cancellous bone. Our study showed that the fracture healing time was 2.92 months, comparable to the value of 2.81 months reported in other studies [15]. While patellar fractures have been managed using only cerclage wires, the strength of fixation provided by this construct is insufficient to enable early mobilization [16]. Pyrford employed the use of cerclage with anterior TBW, which showed superior stability [4]. Our method of fixation with cerclage without K-wire anchorage nullified K-wire-related complications, providing satisfactory outcomes with low complication rates.

Several techniques, such as Lotke's wiring, Magnusson's wiring, and the modified AO technique, are commonly employed to manage patellar fractures. Benjamin et al. showed that among these techniques, the modified AO technique is the most stable one for fixation [17,18]. However, the usage of K-wire has led to complications such as migration, pain, irritation, and possible protrusion out of the skin [8,9]. Hoshino et al. reported that following TBW of patellar fractures using K-wires or cannulated screws, almost 33% of the implants were removed voluntarily. Additionally, fixation using AO K-wires has been linked to twice the risk of implant removal compared to cannulated screws due to symptomatic hardware [10].

A meta-analysis by Dy et al. involving 737 patients reported an infection rate of 3.2% and a nonunion rate of only 1.3%. The reoperation rate, however, was 34% after patella fracture fixation, with the majority of them attributed to K-wire usage in the fixation construct [19]. Our reoperation rate was 2.6%, which is less than what was found in our review of the literature. We found that with this modified technique, problems related to K-wire fixation can be avoided, leading to satisfactory healing rates and good functional outcomes.

The limitations of our study include the small sample size, the lack of a control group, and potential biases in the results. Further research should be conducted to confirm these outcomes using prospective and biomechanics studies.

## Conclusions

Our study presented a novel technique for the surgical management of patellar fractures using cerclage without K-wire anchorage. It can be used in simple as well as in comminuted fractures. This technique offers comparable outcomes to commonly used fixation methods while nullifying K-wire-related complications, resulting in low reoperation rates. Our findings highlight the importance of minimizing hardware usage while achieving desirable functional outcomes in patellar fracture surgery.
